# GPX4 Plays a Crucial Role in Fuzheng Kang’ai Decoction-Induced Non-Small Cell Lung Cancer Cell Ferroptosis

**DOI:** 10.3389/fphar.2022.851680

**Published:** 2022-04-13

**Authors:** Yue-Yang Zhao, Yu-Qi Yang, Hong-Hao Sheng, Qing Tang, Ling Han, Su-Mei Wang, Wan-Yin Wu

**Affiliations:** ^1^ Department of Hematology, Guangdong Provincial Hospital of Chinese Medicine, The Second Clinical Medical College, Guangzhou University of Chinese Medicine, Guangzhou, China; ^2^ Department of Oncology, Clinical and Basic Research Team of TCM Prevention and Treatment of NSCLC, Guangdong Provincial Hospital of Chinese Medicine, The Second Clinical College of Guangzhou University of Chinese Medicine, Guangzhou, China; ^3^ The Postdoctoral Research Station, Guangzhou University of Chinese Medicine, Guangzhou, China; ^4^ The Second Clinical Medical College, The Second Affiliated Hospital of Guangzhou University of Chinese Medicine, Guangzhou University of Chinese Medicine, Guangzhou, China; ^5^ State Key Laboratory of Dampness Syndrome of Chinese Medicine, The Second Affiliated Hospital of Guangzhou University of Chinese Medicine, Guangzhou, China; ^6^ Guangdong Provincial Key Laboratory of Clinical Research on Traditional Chinese Medicine Syndrome, Guangdong-Hong Kong-Macau Joint Lab on Chinese Medicine and Immune Disease Research, Guangzhou University of Chinese Medicine, Guangzhou, China

**Keywords:** FZKA, NSCLC, ferroptosis, GPX4, TCM

## Abstract

**Background:** Fuzheng Kang’ai decoction (FZKA) has been widely used to treat Non-Small Cell Lung Cancer (NSCLC) patients in China for decades, showing definitively curative effects in clinic. Recently, we found that FZKA could induce NSCLC cell ferroptosis, another type of programmed cell death (PCD), which is totally different from cell apoptosis. Therefore, in the present study, we aim to discover the exact mechanism by which FZKA induces NSCLC cell ferroptosis, which is rarely studied in Traditional Chinese Medicine (TCM).

**Methods:** Cell proliferation assay were performed to detect the cell viability. Cell ferroptosis triggered by FZKA was observed by performing lipid peroxidation assay, Fe^2+^ Ions assay, and mitochondrial ultrastructure by transmission electron microscopy. Ferroptosis inhibitors including liproxstatin-1 and UAMC 3203 were used to block ferroptosis. The ratio of GSH/GSSG was done to measure the alteration of oxidative stress. Western blot and qRT-PCR were carried out to detect the expression of solute carrier family 7 member 11 (SLC7A11), solute carrier family 3 member 2 (SLC3A2) and glutathione peroxidase 4 (GPX4) at protein and mRNA levels, respectively. Lentivirus transfection was performed to overexpress GPX4 stably. Animal model was done to verify the effect of FZKA-induced ferroptosis in NSCLC *in vivo* and immunohistochemistry was done to detect the expression of SLC7A11, SLC3A2 and GPX4 at protein level.

**Results:** First of all, *in vitro* experiments confirmed the inhibition effect of FZKA on NSCLC cell growth. We then, for the first time, found that FZKA induced NSCLC cell ferroptosis by increasing lipid peroxidation and cellular Fe^2+^ Ions. Moreover, characteristic morphological changes of NSCLC cell ferroptosis was observed under transmission electron microscopy. Mechanistically, GPX4, as a key inhibitor of lipid peroxidation, was greatly suppressed by FZKA treatment both at protein and mRNA levels. Furthermore, system xc^−^ (SLC7A11 and SLC3A2) were found to be suppressed and a decreased GSH/GSSG ratio was observed at the same time when treated with FZKA. Notably, overexpressing GPX4 reversed the effect of FZKA-induced NSCLC cell ferroptosis significantly. Finally, the above effect was validated using animal model *in vivo*.

**Conclusion:** Our findings conclude that GPX4 plays a crucial role in FZKA-induced NSCLC cell ferroptosis, providing a novel molecular mechanism by which FZKA treats NSCLC.

## Background

The burden of cancer incidence and mortality is rapidly growing worldwide. Lung cancer remained the leading cause of cancer death, with an estimated 1.8 million death in 2020 (Sung et al., 2021). It is a serious threat to human health. NSCLC is the most common type of lung cancer, accounting for about 85% of all lung malignancies (Herbst et al., 2008). Approximately 70% of NSCLC is topically advanced or metastatic at the time of diagnosis (Molina et al., 2008). Until the last decade, the 5-year overall survival rate for patients with metastatic NSCLC was less than 5% (Arbour and Riely, 2019). Improved understanding of the biology of lung cancer had resulted in the development of new biomarker-targeted therapies and led to improvements for patients with advanced or metastatic cancers (Jordan et al., 2017; Melosky et al., 2018; Zhu et al., 2020). Disease progression is unavoidable in the advanced stage, thus additional strategies to extend survival and improve quality of life (QoL) are required. The Chinese herbal medicine (CHM) has been commonly used in cancer treatment as an adjuvant therapy in many countries, especially in China. It has been found that CHM has potential benefit in retarding tumor progression (Wang et al., 2018a; Luo et al., 2019).

FZKA, a formular containing 12 CHMs, has been confirmed to have definite benefit for treating NSCLC patients. In our previous study, we found that FZKA combined with geftinib could prolong progression-free survival (PFS) and reduce the toxic effect, comparing with geftinib alone (Yang et al., 2018). In addition, FZKA could also enhance the disease control rate (DCR) as well as median survival time (MST) of NSCLC patients (Wu et al., 2010; Xiao-Bing Yang et al., 2014). Our further basic research showed that FZKA could inhibit NSCLC cell proliferation and promote cell apoptosis *via* AMPKα/IGFBP1/FOXO3a and STAT3/Bcl-2/Caspase-3 pathways, respectively (Zheng et al., 2016; Wang et al., 2018b). Here in the present study, we, for the first time, found that FZKA could induce NSCLC cell ferroptosis.

Ferroptosis, first described in 2012 (Dixon et al., 2012), is characterized by iron-dependent lipid peroxidation and metabolic constraints (Seibt et al., 2019). The happening of specific lipid peroxidation products directly precedes cellular disintegration and cell death (Angeli et al., 2017). Mechanistically, cysteine availability, glutathione (GSH) biosynthesis and proper functioning of GPX4 are crucial in the process of cell ferroptosis. GPX4 is a key inhibitor of lipid peroxidation. Ferroptotic cell death will be triggered on the condition of GPX4 inhibition. Ferroptosis is characterized by the generation of specific phospholipid hydroperoxides in the presence of catalytically active iron, which is endogenously offset by the system xc^−^/GSH/GPX4 axis (Dixon et al., 2012; Friedmann Angeli et al., 2014; Wan Seok Yang et al., 2014). System xc^−^, composed of SLC7A11 and SLC3A2, is a cystine-glutamate anti-porter (Conrad and Sato, 2012). Therefore, disturbances in any of these protective compartments will result in ferroptotic cell death. In our study, we identified that system xc^−^/GSH/GPX4 axis was involved in FZKA-induced NSCLC cell ferroptosis and GPX4 is the key molecular.

## Materials and Methods

### Fuzheng Kang’ai Decoction

FZKA, containing 12 components, was obtained from Guangdong Kangmei Pharmaceutical Company Ltd. (Guangdong, China), as previously reported (Xiao-Bing Yang et al., 2014). The components of FZKA include *Radix Pseudostellariae* 30 g, *Rhizoma Atractylodis Macrocephalae* 15 g, *Milkvetch Root* 30 g, *Hedyotis Difusa* 30 g, *Solanum Nigrum* 30 g, *Chinese Sage Herb* 30 g, *Indian Iphigenia Bulb* 30 g, *Coix Seed* 30 g, *Akebia Trifoliata Koidz* 30 g, *Snake Bubble Ilicifolius* 30 g, *Curcuma Zedoaria* 15 g, *Licorice* 10 g. For *in vitro* experiments, the granules were dissolved in RPMI-1640 medium to a final concentration of 20 mg/ml and centrifuged at 14,000 rpm for 10 min; the supernatant was then filtered using 0.22 μm filter before use and the pH value of the cultured cells with the media was adjusted to 7.2–7.4 after FZKA addition. For *in vivo* experiments, animals were treated with FZKA by intragastric administration.

### High Performance Liquid Chromatography

The initial batch to batch consistency study was performed using HPLC, as previously reported (Zheng et al., 2016). Briefly, the sample solutions were put into the HPLC system (250 mm × 4.6 mm, 5 μm, ACE, Scotland). The mobile phase consisted of deionized water with 0.1% formic acid (A) and acetonitrile with 0.1% formic acid (B). The gradient elution program was as follows: 5% B at 0–5 min, 5–20% B at 5–10 min, 20–40% B at 10–15 min, 40–95% B at 15–40 min, and 95–100% B at 40–45 min. The flow rate was 1.0 ml/min, and the detection wavelength was set at 280 nm. The injection volume was 10 μl and the column temperature was maintained at 30°C. The efficacy of different batch of FZKA is dependable (Wang et al., 2020).

### Cell Lines, Reagents and Antibodies

NSCLC cell lines including A549, H1299, PC9 and H1650 were obtained from Guangzhou Cellcook Biotech Co. (Guangzhou, China). All cells were grown at 37°C in a humidified 5% CO_2_ and 95% air and cultured in RPMI-1640 medium (Life Technologies, Carlsbad, CA, United States) containing 10% FBS (Gibco, United States) and 0.5% penicillin-streptomycin sulfate (Invitrogen Life Technologies, Carlsbad, CA, United States). Annexin V-FITC Apoptosis Detection Kit and Cell Counting Kit (CCK-8) were purchased from Shanghai Yisheng Biotechnology Co. (Shanghai, China). BODIPY™ 581/591 C11 was obtained from Thermo Fisher Scientific (Waltham, MA). Lentiviral vectors for overexpression constructs were purchased from GeneCopoeia (Rockville, United States). The antibodies were obtained from the following sources: GPX4 (ab125066) and GAPDH (ab9485) were purchased from Abcam (Cambridge, United Kingdom); SLC3A2 (4F2hc/CD98) (47213S), SLC7A11 (12691S), horseradish peroxidase (HRP)-conjugated goat anti-rabbit antibody (7074S), were from Cell Signalling Technology (Danvers, MA); SLC7A11 (bs-6883R) for immunohistochemistry was obtained from Bioss Biological Technology Co. Ltd. (Beijing, China). FerroOrange and GSSG/GSH Quantification Kit were purchased from Dojindo Molecular Technologies Company (Kumamoto, Japan).

### Cell Counting Kit-8 Assay

Cell proliferation was measured by CCK-8 assay according to the manufacturer’s protocol. Briefly, the NSCLC cells were administered with different treatments, and incubated with the CCK-8 reaction solution for 1.5 h. After that, the optical density (OD) values were measured at the wavelength of 450 nm to evaluate cell viability.

### 5-Ethynyl-2′-Deoxyuridine Proliferation Assay

5-ethynyl-2′-deoxyuridine (EdU) proliferation assay was performed to measure cell proliferation. Cells were plated in 96-well plates at a density of 8 × 10^3^ cells/well. After adding FZKA for 24 h, cells were treated with 50 µM EdU (RiboBio, Guangzhou, China) and fixed with 4% paraformaldehyde in PBS for 30 min. After permeabilization with 0.5% TritonX-100 for 10 min, the cells were stained with 1× Apollo reaction reagent. Then the DNA contents were stained with Hoechst 33,342 for 30 min. The photographs were obtained using fluorescence microscope.

### Flow Cytometry of Cell Death Distribution

Cells were treated with FZKA and ferroptosis inhibitors. Annexin V-FITC Apoptosis Detection Kit was used to observe the quadrant distribution of cell death. Both floating and adherent cells were collected and washed 3 times with PBS. Finally, 10 μl Annexin V-FITC and 5 μl PI were added into the cells at room temperature for 15 min. The quadrant distribution of cell death was measured using flow cytometry with the acquisition criteria of 10,000 events for each sample.

### Transmission Electron Microscopy

The mitochondrial ultrastructure was observed by transmission electron microscopy (TEM). 2 × 10^6^ cells were seeded into 100 mm cell culture dishes and exposed to FZKA decoction and erastin for 10 h, respectively. After that, cells were collected, washed three times with PBS, and fixed with 2.5% glutaraldehyde. Samples were then pretreated according to standard procedures, including staining, dehydration, embedding, and slicing to obtain ultra-thin sections. During the analysis, images were acquired using a HITACHIH-7650 transmission electron microscope (Hitachi, Tokyo, Japan).

### Lipid Peroxidation Measurement

C11-BODIPY (10 μM) was added to FZKA treated or untreated cells for 0.5 h, then cells were collected by trypsin. Oxidation of the poly-unsaturated butadienyl portion of C11-BODIPY resulted in a shift of the fluorescence emission peak from ∼590 to ∼510 nm. Samples were analyzed using flow cytometry (Exc: 488 nm, Em: 510 nm) after washing twice with PBS, and the results were analyzed by NovoExpress software.

### Detection of Cellular Fe^2+^ Ions Generation

To clarify Fe^2+^ ions generation via the nanoparticles in cells, FerroOrange (1 μM, an intracellular Fe^2+^ ions probe, Ex: 543 nm, Em: 580 nm) dispersed in serum-free medium was added to the cells, and cells were incubated for 30 min in a 37°C incubator. Cells were then collected by trypsin. Finally, the fluorescence of cells were captured using flow cytometry after washing twice with PBS.

### Lentivirus Transfection

Lentiviral vectors including GPX4 (CS-M0369-Lv105) and Negative Control (EX-NEG-Lv105) were purchased from GeneCopoeia. For lentivirus production, HEK293T packaging cells were transfected with 10 μg lentiviral vectors using the calcium phosphate method. After 48 h of incubation, the viral supernatant was collected and filtered. NSCLC cells were incubated overnight with the viral supernatant and supplemented with 10 μg/ml polybrene. Puromycin at a dose of 2 μg/ml was used to select the cell line overexpressing GPX4 stably.

### Western Blot Analysis

Western blot was conducted as previously reported (Wang et al., 2018b). Briefly, the cells were harvested, washed and lysed with 1 × RIPA buffer, and their protein concentrations were measured using Bradford method. SDS-PAGE was used to separate the protein in each sample. Proteins were transferred from gel to membrane. Then, the membrane was blocked and incubated with indicated primary antibodies. The blots were rinsed before probed with secondary antibodies. The reactive bands were visualized by ECL and scanned using the Bio-Rad ChemiDoc XRS + Chemiluminescence imaging system (Bio-Rad, Hercules, CA, United States). All the results were analyzed by ImageJ software.

### Quantitative Real-Time PCR

Total RNA was isolated using Trizol (Invitrogen, CA, United States). Transcriptor first strand cDNA synthesis kit (Roche, Basel, Switzerland) was used to convert RNAs to cDNAs. And FS essential DNA green master (Roche, Basel, Switzerland) was used to perform qRT-PCR. Complementary DNA from various cell samples was amplified with specific primers. GPX4: 5′-AGT​GAG​GCA​AGA​CCG​AAG​T-3′ and 5′- AAC​TGG​TTA​CAC​GGG​AAG​G-3′; GAPDH: 5′- GAACGGGAAGCTCACTGG -3′ and 5′- GCC​TGC​TTC​ACC​ACC​TTC​T -3′. Data were analyzed with 2^−ΔΔCt^ for relative changes in gene expression.

### Glutathione/Glutathione Disulfide Assay

The intracellular level of GSH and GSSG (glutathione disulfide) were performed by GSSG/GSH Quantification Kit (Dojindo, Kumamoto, Japan), following the manufacturer’s instruction. The concentration of total glutathione or GSSG was calculated via standard curve. GSH level was calculated as: GSH = (total glutathione-GSSG) × 2. The ratio of GSH/GSSG was calculated as (GSH)/(GSSG).

### Animal Model

All animal experiments were approved by the Ethics Committee of Guangdong Provincial Hospital of Chinese Medicine (2020079). A total of 1.0 × 10^6^ A549 cells were subcutaneously injected into the right flank of the athymic BALB/c nude mice (aged 4–6 weeks, weight 18–20 g, female; Vital River, Beijing, China). When the tumor mass became palpable (at day 5 after injection), the mice were randomly divided into three groups: control, FZKA (31 g/kg) and combination with FZKA and liproxstatin-1(30 mg/kg). Tumors were measured every 5 days with digital calipers. The tumor volume (in mm^3^) was calculated using the formula: Volume=(L×W^2^)/2. Mice were sacrificed around day 25 after injection, when some of the tumors reached the size limit set by the institutional animal care and use committee. Tumors were weighed after careful resection.

### Immunohistochemistry

The protein levels of GPX4, SLC7A11 and SLC3A2 expression were detected immunohistochemically on paraffin-embedded xenograft tumor tissue sections. Briefly, sections were treated with 10 mM sodium citrate buffer (pH 6.0) for heat-induced retrieval of the antigen and immersed in 3% hydrogen peroxide solution to inhibit endogenous peroxidase activity, followed by incubation of the sections in 5% bovine serum albumin to block nonspecific binding. The sections were incubated with primary antibodies against GPX4 (1:250), SLC7A11 (1:100) and SLC3A2 (1:100) at 4°C overnight and then incubated with biotinylated secondary antibody followed by the Liquid DAB Substrate Chromogen System according to the manufacturer’s instructions. Protein expression level was evaluated by counting at least 500 tumor cells in at least five representative high-power fields. The percentage of positive tumor cells and the staining intensity were multiplied to produce a weighted score for each case (Lu et al., 1998).

### Statistical Analysis

Statistical analysis was performed using the SPSS statistical software. Statistical evaluation for data analysis used Student’s t-test when there were only two groups (two sided) and differences between groups were assessed by one-way ANOVA. All data are reported as Mean ± SD. Differences between groups were considered significant statistically when *p* ≤0.05.

## Results

### Non-Small Cell Lung Cancer Cell Growth was Inhibited by Fuzheng Kang’ai Decoction *in vitro*

**FIGURE 1:**
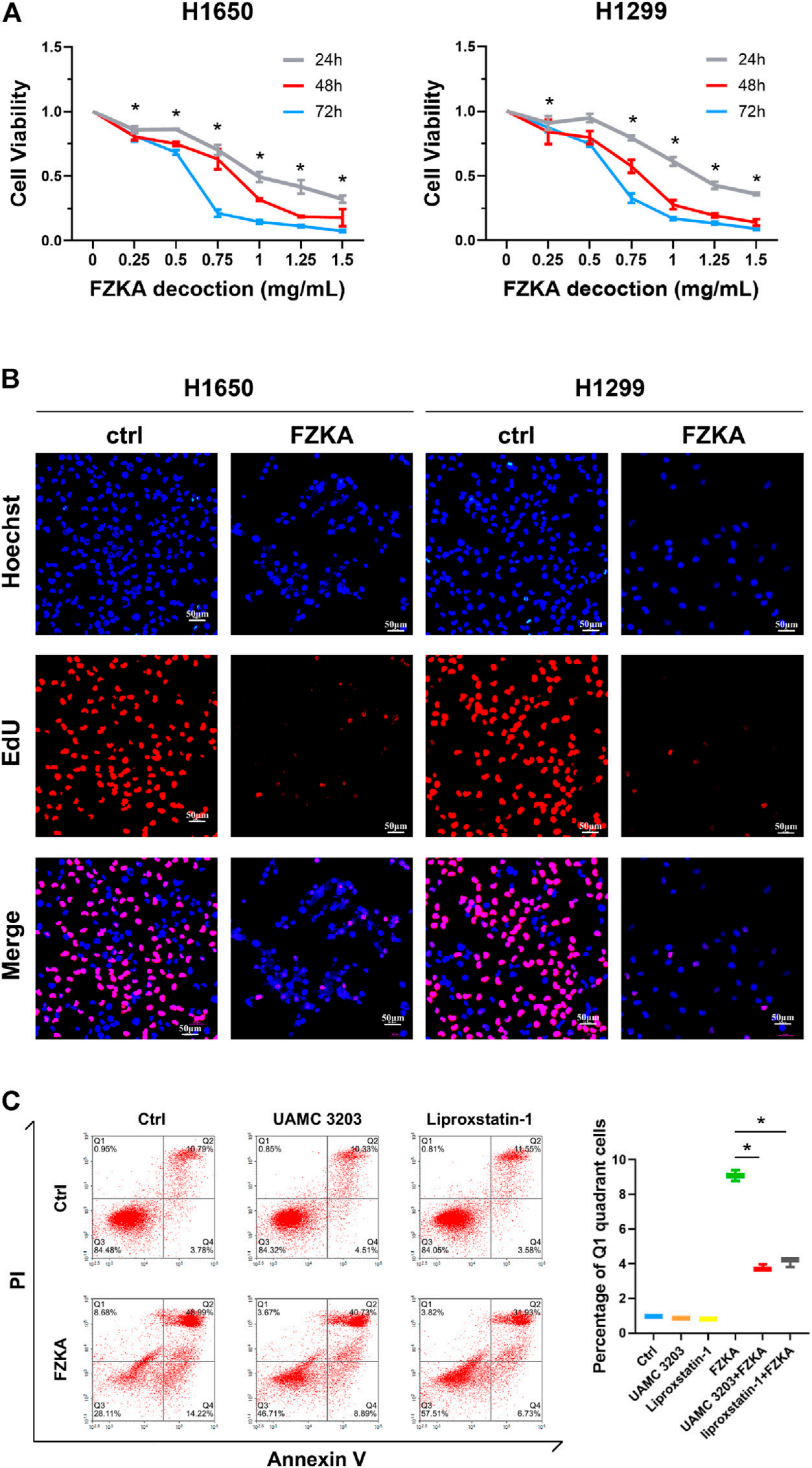
FZKA inhibited the growth of NSCLC cells *in vitro*. **(A)**, H1650 and H1299 cells were treated with different concentrations of FZKA for up to 72 h. The cells were collected and processed for CCK-8 assay as described in the Materials and Methods section, *p < 0.05. **(B)**, H1650 and H1299 cells were treated with FZKA (1 mg/ml) for 24 h, followed by EdU proliferation assay. **(C)**. Cultured A549 cells were treated with FZKA (1.5 mg/ml), in the presence and absence of ferroptosis inhibitors. Cell were stained with Annexin V and PI and analyzed by flow cytometry, *p < 0.05.

Our previous studies have shown that FZKA inhibited the growth of NSCLC cell lines including A549, PC9, and H1975 cells (Wang et al., 2020; Zheng et al., 2019). In the present study, we further observed the effect of FZKA on NSCLC cell growth inhibition in another NSCLC cell types including H1650 and H1299 using CCK-8 assay. We reconfirmed that FZKA decreased H1650 and H1299 cell viability in a dose-and time-dependent manner (Figure 1A). Similar findings were also demonstrated by EdU proliferation assay, which detects EdU incorporated into cellular DNA during cell proliferation (Figure 1B). Intriguingly, we found that blocking ferroptosis by UAMC 3203 and liproxstatin-1 could significantly reversed changes in the quadrants of Annexin V-/PI+ (Q1) and Annexin V+/PI+ (Figure 1C). The cells in Q1 quadrant in the top left had been supposed as non-apoptotic cells, and ferroptotic cell death was included in Q1 quadrant (Gai et al., 2020). The data showed that inhibiting ferroptosis could decrease the percent of Q1 induced by FZKA, which suggests that ferroptosis plays an important role in the inhibition effect of FZKA in NSCLC cells and FZKA may promote ferroptosis in NSCLC cells.

### Non-Small Cell Lung Cancer Cell Ferroptosis was Induced by Fuzheng Kang’ai decoction

**FIGURE 2:**
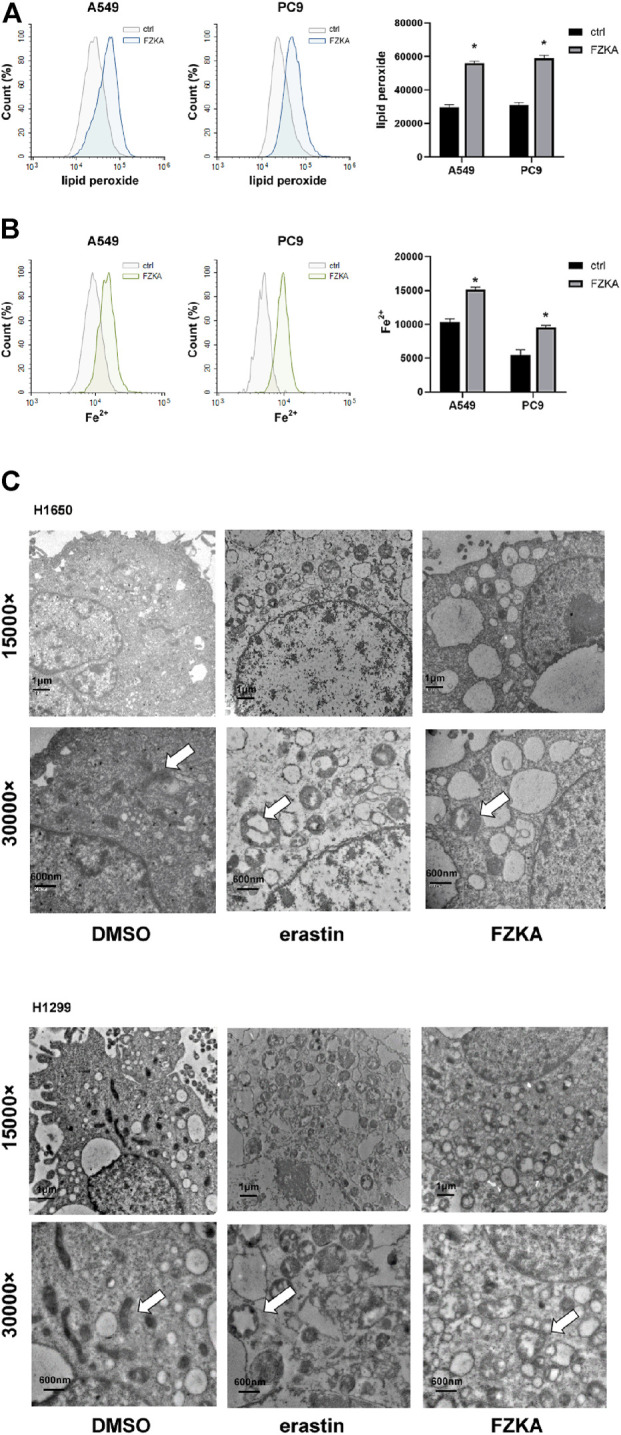
FZKA induced ferroptosis in NSCLC cells by FCM and TEM. **(A,B)**, Cells were stained with C11-BODIPY (10 μM) or FerroOrange (1 μM) for 30 min, the level of lipid peroxidation and Fe^2+^ Ions was detected by flow cytometry, *p < 0.05. **(C)**, Transmission electron microscopy of H1650 and H1299 cells treated with DMSO (10 h), erastin (ferroptosis inducer, 10 µM, 10 h), FZKA (1 mg/ml, 10 h). The mitochondria appeared to be changed including swollen, cristae loss and mitochondira vacuolization in H1650 and H1299 cells. Scale bar represents 1 µM and 600 nm.

To identify whether NSCLC cell ferroptosis was induced by FZKA treatment, lipid peroxidation and intracellular-free iron, as two key characteristics of cell ferroptosis, were then detected in NSCLC cells after treatment with FZKA (Lei et al., 2019). C11-BODIPY was used as a lipid peroxidation probe in mammalian cells (Ortega Ferrusola et al., 2009). The intracellular labile Fe (II) levels in the living cells were measured by FerroOrange (Hirayama et al., 2020). The results showed that FZKA increased the levels of lipid peroxidation (Figure 2A) and intracellular-free iron (Figure 2B) in A549 and PC9 cells. The same results were also observed in H1299 and H1650 cells (Supplementary Figure S1). Moreover, the characteristic changes of ferroptosis on mitochondria, including swollen, decreased cristae, mitochondria vacuolization and increased membrane density, were further observed under TEM in NSCLC cells (Figure 2C, Supplementary Figure S2).

### Blocking Ferroptosis Reversed the Inhibition Effect of Fuzheng Kang’ai decoction on Non-Small Cell Lung Cancer Cells

**FIGURE 3:**
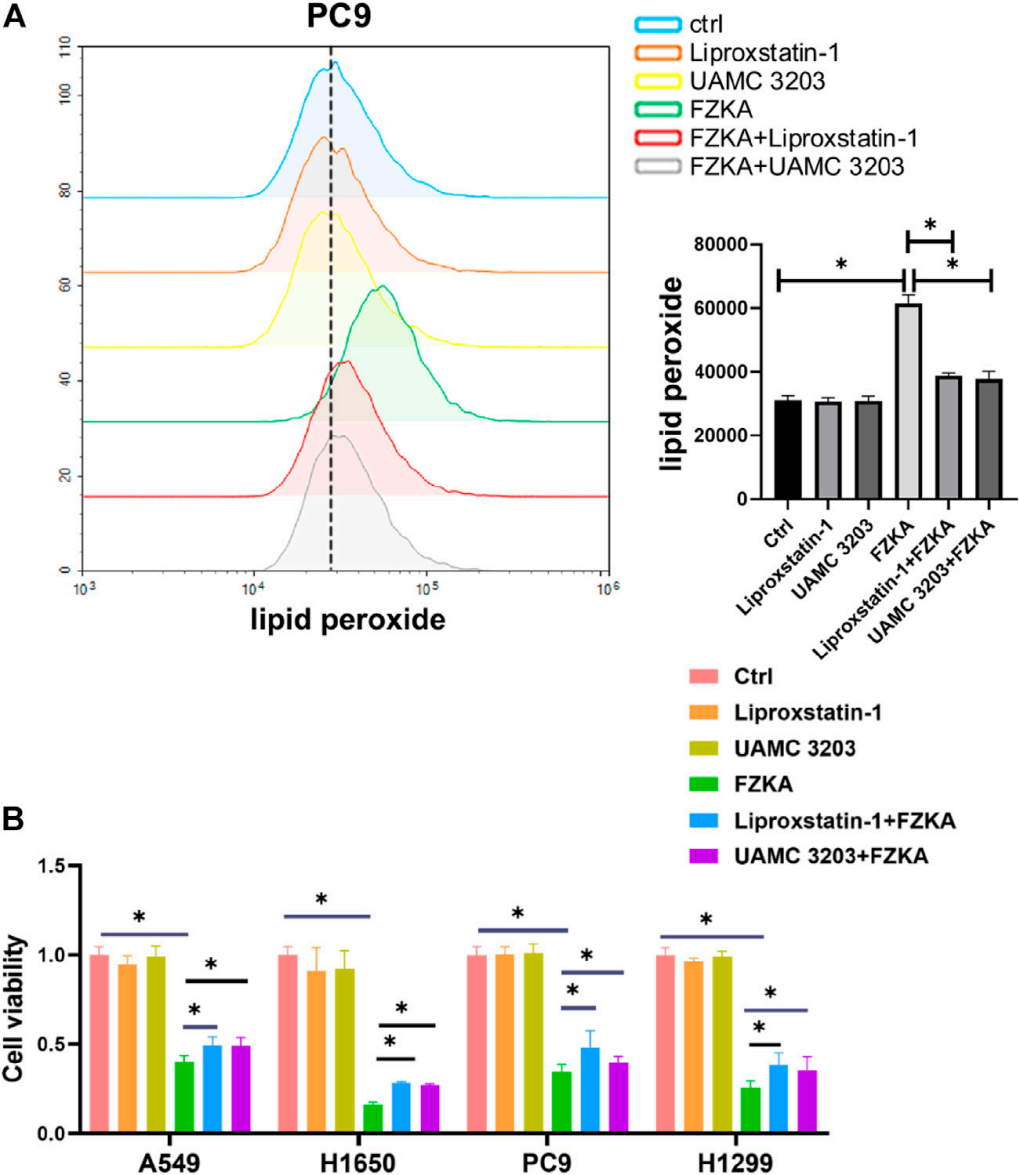
Ferroptosis inhibitors including liproxstatin-1 and UAMC 3203 reversed the effect of FZKA. A, PC9 cells were treated as **(A)** FZKA (1.5 mg/ml), UAMC3203 (25 nM) or liproxstatin-1 (200 nM) for 24 h, and stained with BODIPY™ 581/591 C11 (10 μM) for 30 min. The level of lipid peroxidation was detected by flow cytometry. Each point represents the mean ± SEM, *n* = 3, *p <0.05. **(B)**, Cultured NSCLC cells were seeded in 96 well plate, FZKA (1.5 mg/ml in A549 and PC9 cells, 1 mg/ml in H1650 and H1299 cells), with UAMC3203 (25 nM), or liproxstatin-1 (200 nM) for 24 h. Cell viability was detected by CCK-8 assay, *p < 0.05.

To further observe the role of ferroptosis in FZKA-treated NSCLC cells, two ferroptosis inhibitors including UAMC 3203 and liproxstatin-1 were applied to block ferroptosis. As shown in Figure 3A and Supplementary Figure S3, the FZKA-induced elevation of lipid peroxidation was almost reversed by treating with UAMC 3203 and liproxstatin-1. Our further data showed that blocking ferroptosis remarkedly reversed the inhibition effect of FZKA on NSCLC cell lines (A549, PC9, H1650 and H1299), as shown in the Figure 3B. These results indicated that ferroptosis plays a vital role in FZKA treated NSCLC cells.

### Glutathione Peroxidase 4 was Significantly Suppressed by Fuzheng Kang’ai Decoction in Non-Small Cell Lung Cancer Cells

**FIGURE 4:**
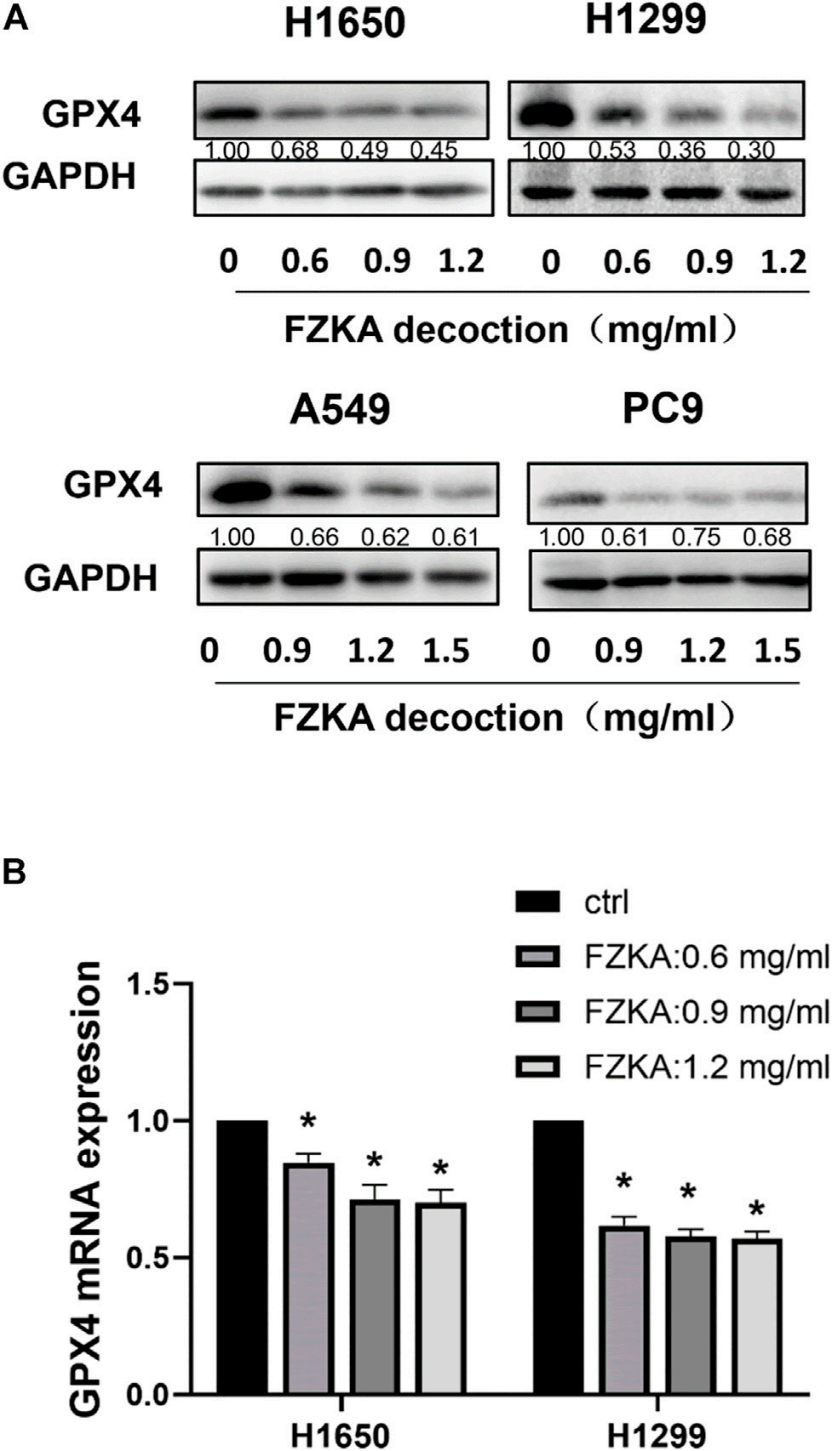
FZKA downregulated the GPX4 expression in NSCLC cells at both protein and mRNA levels. **(A)**, The protein expression levels of GPX4 were detected by Western blot. **(B)**, NSCLC cells were treated with different concentrations of FZKA for 24 h. The mRNA expression of GPX4 were detected by qPCR. Each point represents the mean ± SEM, *n* = 3. *p < 0.05.

GPX4 is a key inhibitor of lipid peroxidation and ferroptosis. The down-regulation of GPX4 could directly or indirectly trigger ferroptosis as a result of lipid peroxidation inhibition. We detected the expressions of GPX4 at protein and mRNA level after treatment with FZKA. Our data found that the protein level of GPX4 was significantly decreased following the application of FZKA in NSCLC cells (Figure 4A). Meanwhile, the mRNA level of GPX4 were also decreased by treating with FZKA (Figure 4B). The above data indicated that GPX4 might be a main molecular in the FZKA-induced NSCLC cell ferroptosis process.

### System xc^−^/Glutathione Axis was Involved in Fuzheng Kang’ai Decoction-Induced Non-Small Cell Lung Cancer Cell Ferroptosis

**FIGURE 5:**
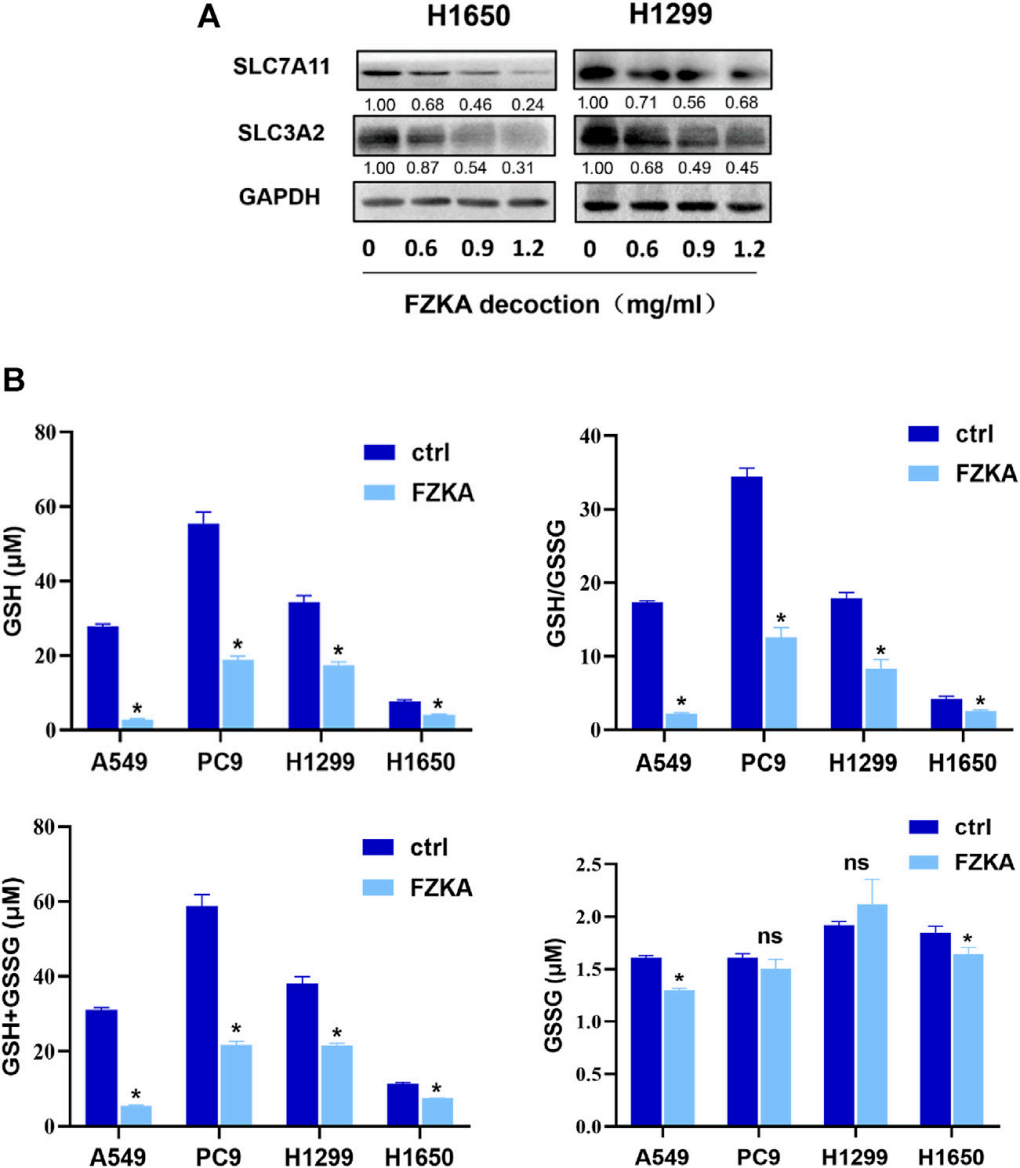
FZKA decreased the ratio of GSH/GSSG and expression of system xc^−^. **(A)**, The protein expression levels of SLC7A11 and SLC3A2 were detected by Western blot. **(B)**, the levels of GSH and GSSG were measured by GSH and GSSG Assay kit. Each point represents the mean ± SEM, *n* = 3. *p < 0.05.

The cystine-glutamate antiporter system xc^−^, which is composed of the subunits SLC7A11 and SLC3A2, plays a protective role against cell ferroptosis (Cui et al., 2018). We revealed that the protein levels of SLC7A11 and SLC3A2 were obviously decreased following the application of FZKA in a dose-dependent manner in H1650 and H1299 cells (Figure 5A). Glutathione is a tripeptide, that is, derived from cysteine, glutamate, and glycine, among which cysteine is the rate-limiting precursor. As expected, the amount of GSH was significantly decreased by FZKA. GSH is highly reactive with lipid ROS, and their reaction generates glutathione disulfide (GSSG). A reduced ratio of GSH/GSSG is considered to be a marker of oxidative stress. In our study, we found that FZKA reduced the ratio of GSH/GSSG significantly (Figure 5B). System xc^−^ and GSH are at the upstream of GPX4, therefore, our data showed that system xc^−^/GSH/GPX4 axis plays an important role in FZKA-induced NSCLC cell ferroptosis.

### Over-Expressing Glutathione Peroxidase 4 Reversed the Effect of Fuzheng Kang’ai Decoction-Induced Cell Ferroptosis

**FIGURE 6:**
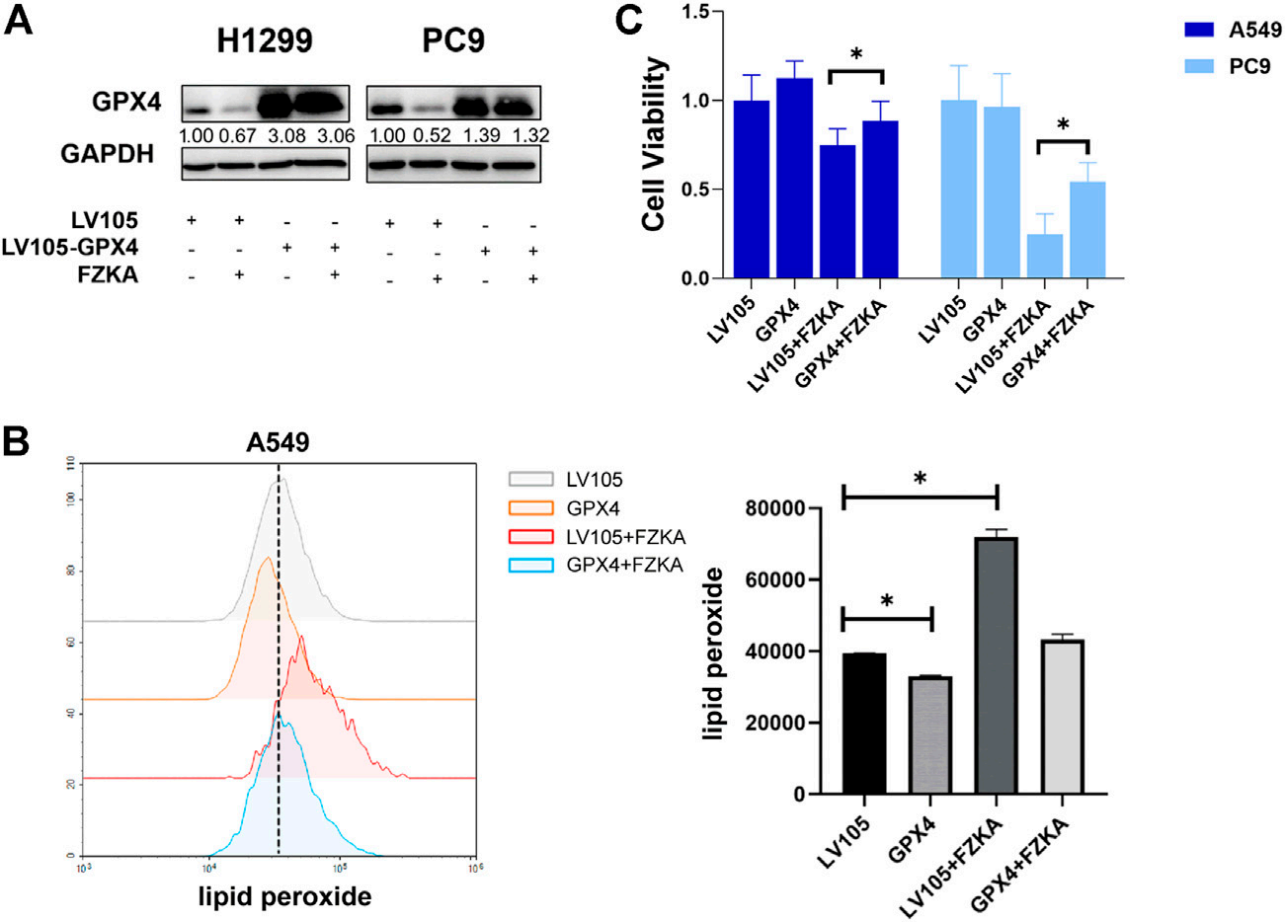
Overexpression of GPX4 reversed the efficacy of FZKA. **(A)**, Cultured H1299 and PC9 cells were transfected with negative control and GPX4 lentiviral vectors, then treated with or without FZKA for 24 h. The expression of GPX4 was detected by western blot. **(B)**, lipid peroxidation assay was performed in A549 cell after treatment with FZKA or/and GPX4 lentivirus. **(C)**, Cells were transfected and treated with FZKA, and CCK-8 assay were then conducted. Each point represents the mean ± SEM, *n* = 3. *p < 0.05.

Since GPX4 plays a crucial role in the process of cell ferroptosis, we further confirmed that GPX4′s role in FZKA-induced NSCLC ferroptosis. We then over-expressed GPX4 in H1299 and PC9 cells by transfecting lentivirus (Figure 6A). As expected, the induced effect of NSCLC ferroptosis by FZKA was substantially decreased following GPX4 overexpression as shown by lipid peroxidation assay (Figure 6B and Supplementary Figure S4). Interestingly, NSCLC cell viability inhibition by FZKA was also partially reversed when over-expressed GPX4 (Figure 6C). This data further confirmed that GPX4 contributes to the effect of FZKA-induced NSCLC cell ferroptosis and FZKA-suppressed NSCLC cell growth, suggesting that GPX4 plays a crucial role in FZKA-treated NSCLC cells.

### Fuzheng Kang’ai Decoction Inhibited Non-Small Cell Lung Cancer Tumor Growth by Inducing Ferroptosis *in vivo*

**FIGURE 7:**
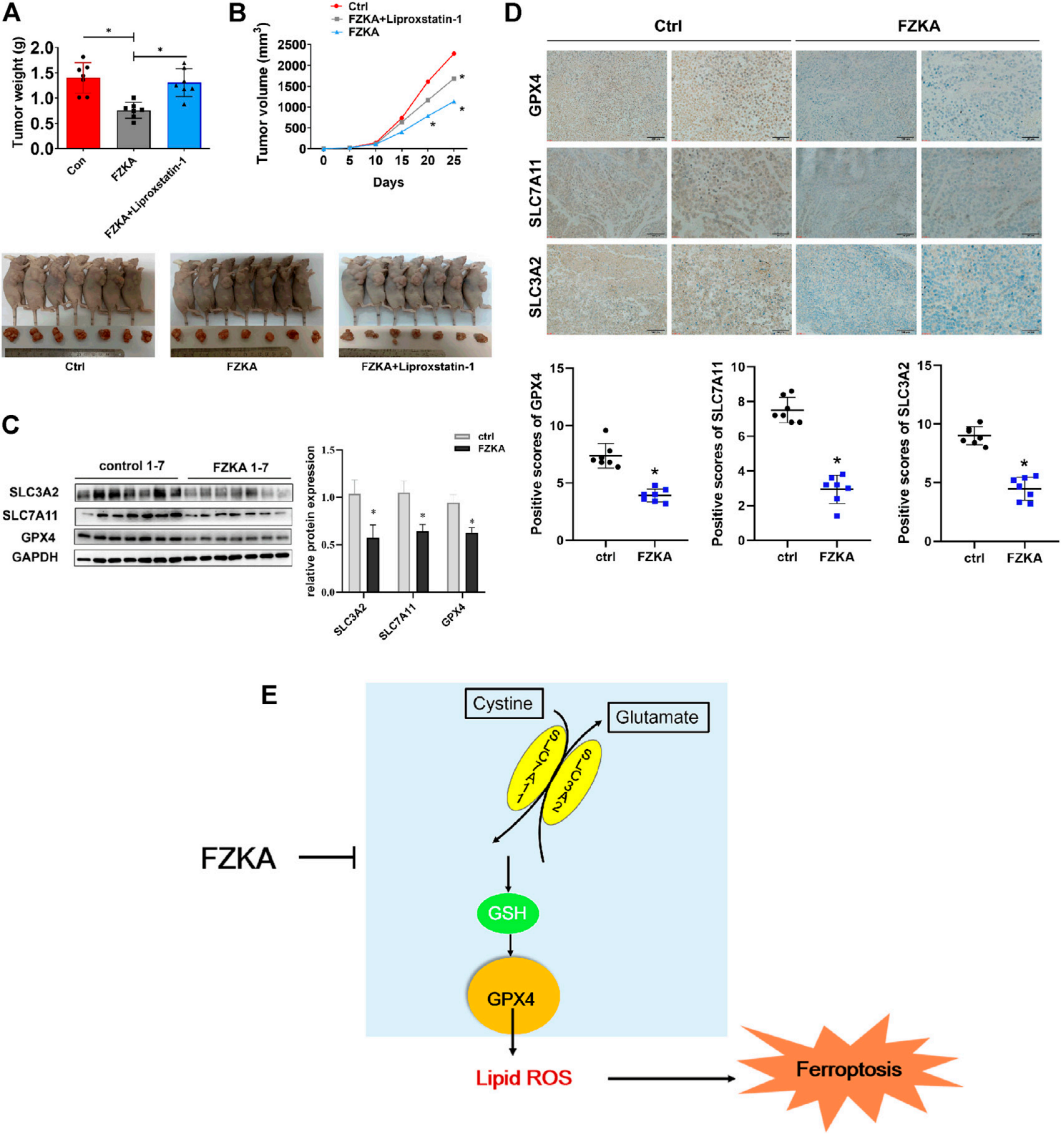
Validation of FZKA-induced NSCLC cell ferroptosis *in vivo*. **(A)**, Mice tumor photograph and tumor weight was showed. Data represents Mean ± SEM, *n* = 7. *p < 0.05. **(B)**, Tumor volume in each group was showed. Data represents Mean ± SEM, *n* = 7. *p < 0.05. **(C)**, Western blot analyses of GPX4, SLC7A11 and SLC3A2 expression from tumor tissues. Data represents Mean ± SEM, *n* = 7. *p < 0.05. **(D)**, Immunohistochemistry was carried out to measure the expression of GPX4, SLC7A11 and SLC3A2 in mice tumor tissues. Data represents Mean ± SEM, *n* = 7. *p < 0.05. **(E)**, The diagram showing FZKA induced NSCLC cell ferroptosis through system xc^−^/GSH/GPX4 axis, and, importantly, GPX4 is the crucial molecular in the process. Finally, inhibition of GPX4 by FZKA leads to NSCLC cell ferroptosis.

To validate the effect of FZKA-induced NSCLC cell ferroptosis *in vivo*, we constructed NSCLC cell xenograft model. As shown in Figures 7A,B, mice tumor growth was obviously inhibited by FZKA treatment undoubtedly. Notably, when cell ferroptosis was blocked by liproxstatin-1, the inhibition effect of tumor growth by FZKA was significantly rescued. Then system xc^−^ and GPX4 were detected by Western blot and immunohistochemistry. As expected, the data was consistent with *in vitro* results showing downregulated expression of system xc^−^ and GPX4 in the FZKA-treated group (Figures 7C,D). Totally, our xenograft model data reconfirmed the effect of FZKA-induced NSCLC cell ferroptosis and system xc^−^/GPX4 axis plays a crucial in the process.

## Discussion

Ferroptosis, as another type of PCD, is entirely different from cell apoptosis, necroptosis, autophagic cell death and other forms of regulated necrotic cell death. The ferroptosis-induced cell death is characterized by iron-dependent lipid peroxidation (Dixon et al., 2012; Seibt et al., 2019). Ferroptotic cell death was considered to locate at Q1 quadrant by flow cytometry of Annexin V/PI staining (Gai et al., 2020). In our study, we found that blocking ferroptosis *via* ferroptosis inhibitors could reversed Q1 quadrant after FZKA treatment, indicating the important role of ferroptosis in FZKA-treated NSCLC cells. Since lipid peroxidation and intracellular-free iron are two key characteristics of cell ferroptosis (Lei et al., 2019), we then detected the lipid peroxidation and intracellular-free iron in NSCLC cells after treatment with FZKA. Our results showed that FZKA could increase lipid peroxidation and intracellular-free iron, indicating that FZKA might have the ability to induce NSCLC cell ferroptosis. We then observed characteristic changes on mitochondria of ferroptosis using TEM in NSCLC cells when treated with FZKA. Treatment with FZKA resulted in swollen mitochondria with fractured cristae and increased membrane density, which is consistent with erastin, a ferroptosis inducer. Therefore, our data provided solid evidences that FZKA induces NSCLC cell ferroptosis.

Early research indicated the primary role of GPX4 in protecting against oxidative damage (Imai et al., 1996; Yagi et al., 1996). Cells with GPX4 overexpression are resistant to lipid hydroperoxide-triggered cell death (Geiger et al., 1991). Later, more studies provided evidences that silencing GPX4 could invariably cause ferroptosis (Maiorino et al., 2018). Some researches reported that GPX4 was decreased at protein levels, resulting in cell ferroptosis (Lou et al., 2021; Zhang et al., 2021). In our study, the expression of GPX4 at the protein and mRNA levels were significantly suppressed by FZKA in NSCLC cells. Disturbances in any of these protective compartments including system xc^−^ and GSH biosynthesis, upstream of GPX4, might result in ferroptotic cell death (Lee et al., 2021). Our results showed that FZKA decreased the expression of SLC7A11 and SLC3A2 in NSCLC cells. And the ratio of GSH/GSSG was also suppressed in FZKA-treated group. Most importantly, when we over-expressed GPX4, it could reverse NSCLC cell ferroptosis induced by FZKA. And cell viability inhibition effect by FZKA was also partially reversed by over-expressing GPX4 at the same time. Therefore, our data indicated the critical role of GPX4 in the induction of NSCLC cell ferroptosis by FZKA. Our findings provide a valid evidence that FZKA might function as a GPX4 inhibitor in treating NSCLC patients, and system xc^−^/GSH/GPX4 axis was involved in the process.

## Conclusion

In our study, we investigated the effect of FZKA on NSCLC cell ferroptosis both *in intro* and *in vivo*. Our results showed that FZKA induces ferroptosis by suppressing GPX4 in NSCLC. We provides solid evidences to clarify why FZKA benefits NSCLC patients in clinic.

## Data Availability

The datasets presented in this study can be found in online repositories. The names of the repository/repositories and accession number(s) can be found in the article/Supplementary Material.

## Abbreviations

FZKA, Fuzheng Kang’ai decoction; NSCLC, Non-Small Cell Lung Cancer; PCD, programmed cell death; TCM, Traditional Chinese Medicine; CCK-8, Cell counting kit-8; EdU, 5-ethynyl-2′-deoxyuridine; SLC7A11, solute carrier family 7 member 11; SLC3A2, solute carrier family 3 member 2; GPX4, glutathione peroxidase 4; QoL, quality of life; CHM, Chinese herbal medicine; PFS, progression-free survival; DCR, disease control rate; MST, median survival time; GSH, glutathione; TEM, transmission electron microscopy; HRP, horseradish peroxidase; OD, optical density; qRT, Quantitative real-time; GSH, glutathione; GSSG, glutathione disulfide.
