# Supplementary figures and images for: GPX4 Plays a Crucial Role in Fuzheng Kang’ai Decoction-Induced Non-Small Cell Lung Cancer Cell Ferroptosis
Source: Front Pharmacol. 2022 Apr 13;13:851680. doi: 10.3389/fphar.2022.851680 (PMC9043103; doi:10.3389/fphar.2022.851680)

Supplementary Figures

Figure S1

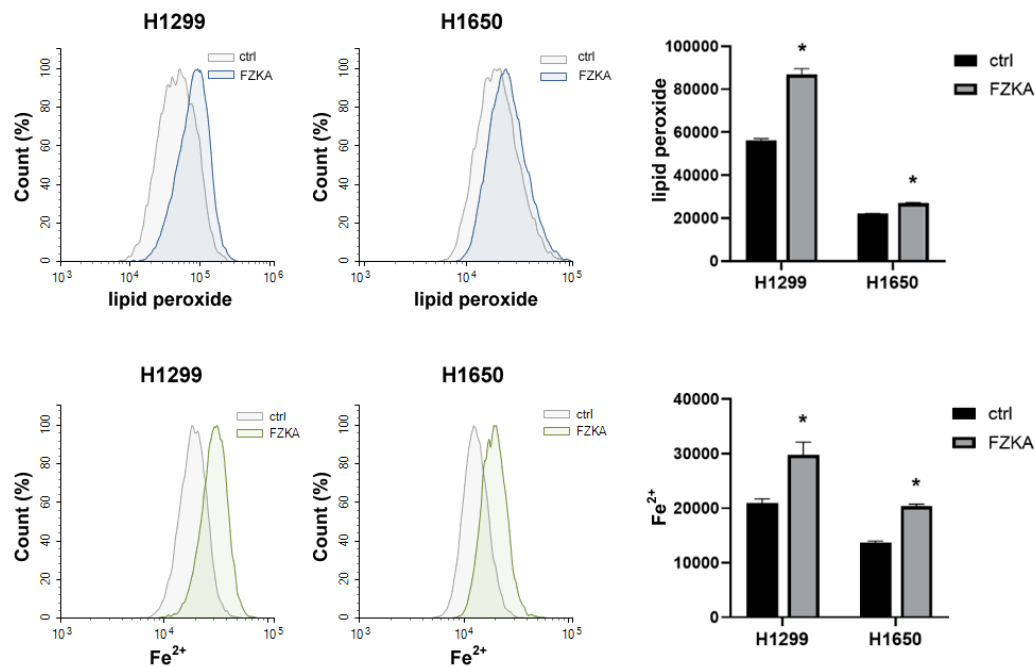

Figure S2

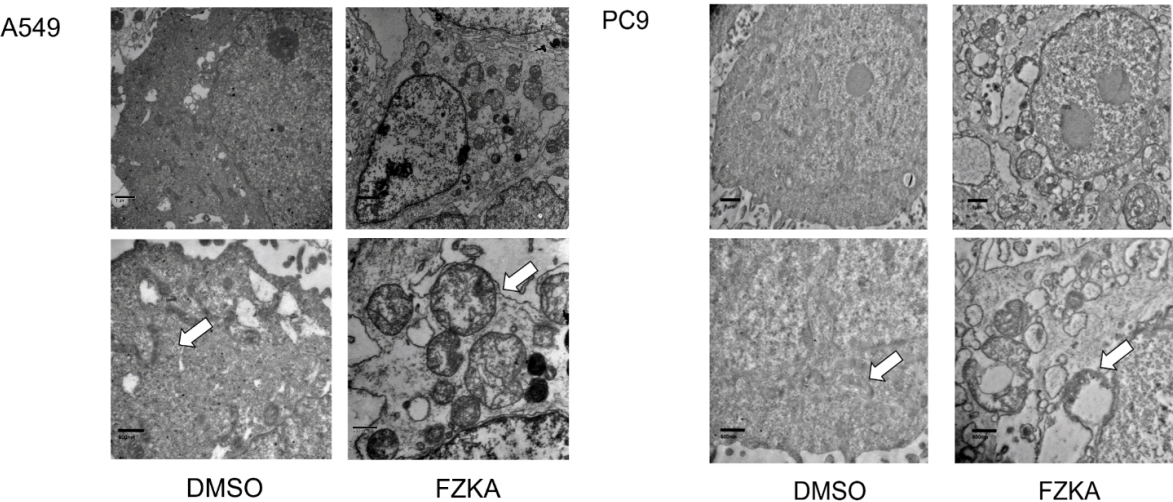

Figure S3

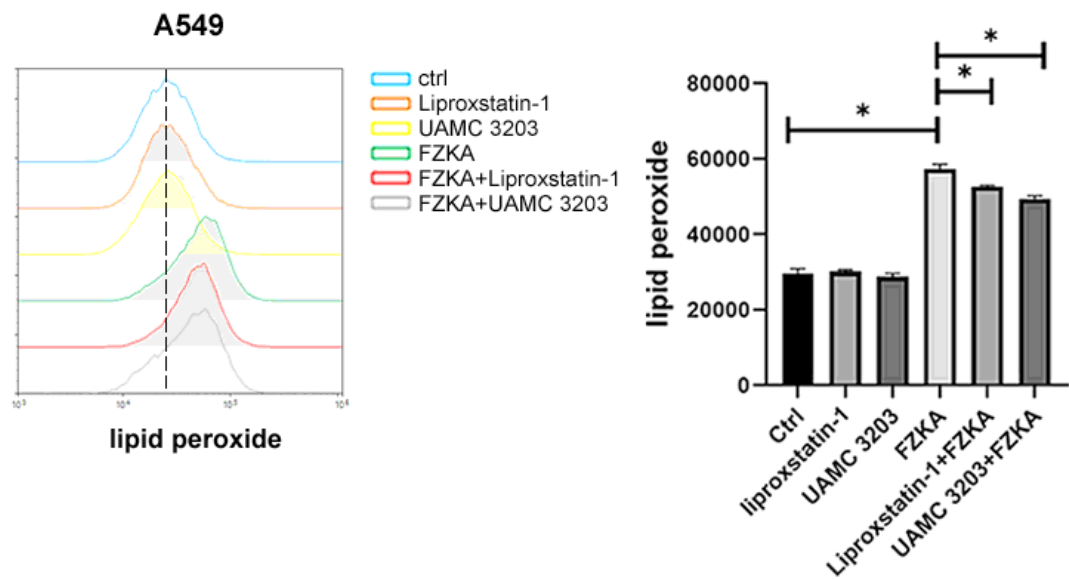

Figure S4

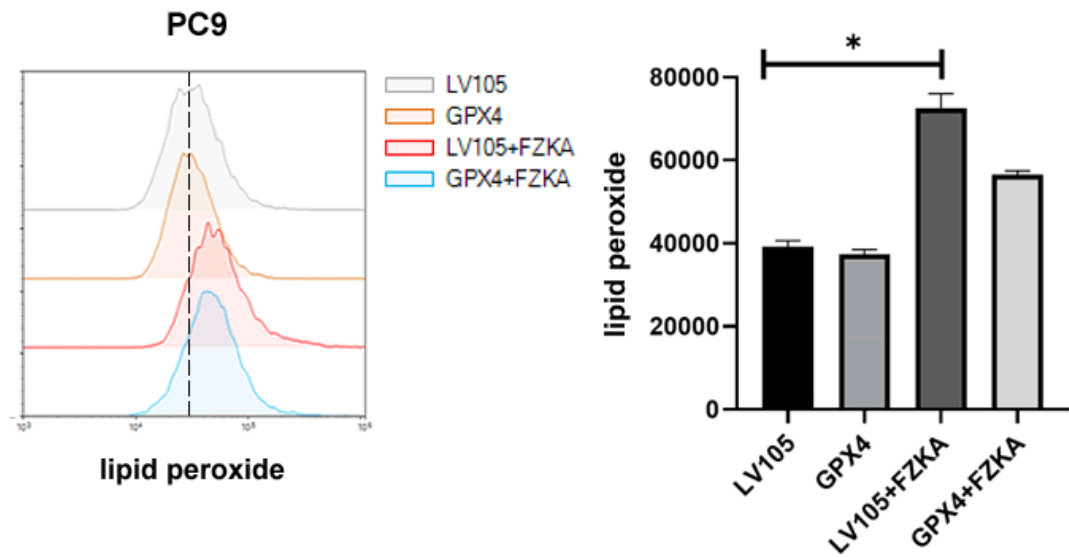

Supplement: Supplementary file 1 [file DataSheet1.PDF]
