# Supplementary material for: GPX4 Plays a Crucial Role in Fuzheng Kang’ai Decoction-Induced Non-Small Cell Lung Cancer Cell Ferroptosis
Source: Front Pharmacol. 2022 Apr 13;13:851680. doi: 10.3389/fphar.2022.851680 (PMC9043103; doi:10.3389/fphar.2022.851680)
Supplement: Supplementary file 2 [file DataSheet2.docx]

**GPX4 plays a crucial role in Fuzheng Kang’ai decoction-induced Non-Small Cell Lung Cancer cell ferroptosis**

Yue-Yang Zhao^1,2,3#^, Yu-Qi Yang^4^*^#^*, Hong-Hao Sheng^2,5,6 #^, Qing Tang^2,5,6^, Ling Han^5,6*^, Su-Mei Wang^2,5,6*^, Wan-Yin Wu^2,5,6*^

^1^ Department of Hematology, Guangdong Provincial Hospital of Chinese Medicine, The Second Clinical Medical College, Guangzhou University of Chinese Medicine, Guangzhou, Guangdong 510120, China

^2^ Department of Oncology, Clinical and Basic Research Team of TCM Prevention and Treatment of NSCLC, The Second Clinical College of Guangzhou University of Chinese Medicine, Guangdong Provincial Hospital of Chinese Medicine, Guangzhou, Guangdong 510120, China

^3^ The Postdoctoral Research Station, Guangzhou University of Chinese Medicine, Guangzhou, Guangdong 510120, China

^4^ The Second Clinical Medical College, Guangzhou University of Chinese Medicine, The Second Affiliated Hospital of Guangzhou University of Chinese Medicine; Guangzhou, Guangdong 510120, China

^5^ State Key laboratory of Dampness Syndrome of Chinese Medicine, The Second Affiliated Hospital of Guangzhou University of Chinese Medicine, Guangzhou, Guangdong 510120, China

^6^ Guangdong Provincial Key Laboratory of Clinical Research on Traditional Chinese Medicine Syndrome; Guangdong-Hong Kong-Macau Joint Lab on Chinese Medicine and Immune Disease Research, Guangzhou University of Chinese Medicine, Guangzhou, Guangdong 510120, P.R. China

^#^ These authors contributed equally.

* Correspondence: linghan99@gzucm.edu.cn; wangsumei@gzucm.edu.cn; wwanyin@gzucm.edu.cn.

Original data links:

(1) [https://www.jianguoyun.com/p/DQl2QuYQ_aKYChjC5KcE](https://www.jianguoyun.com/p/DQl2QuYQ_aKYChjC5KcE)

(2) [https://www.jianguoyun.com/p/DRQYde0Q_aKYChjD5KcE](https://www.jianguoyun.com/p/DRQYde0Q_aKYChjD5KcE)

(3) [https://www.jianguoyun.com/p/DVHDgLsQ_aKYChii5KcE](https://www.jianguoyun.com/p/DVHDgLsQ_aKYChii5KcE)
